# Watching Subtitled Films Can Help Learning Foreign Languages

**DOI:** 10.1371/journal.pone.0158409

**Published:** 2016-06-29

**Authors:** J. Birulés-Muntané, S. Soto-Faraco

**Affiliations:** 1 Department de Tecnologies de la Informació i les Comunicacions, Cognition and Brain Centre, Universitat Pompeu Fabra, Barcelona, Spain; 2 Institució Catalana de Recerca i Estudis Avançats (ICREA), Barcelona, Spain; The University of Nottingham, UNITED KINGDOM

## Abstract

Watching English-spoken films with subtitles is becoming increasingly popular throughout the world. One reason for this trend is the assumption that perceptual learning of the sounds of a foreign language, English, will improve perception skills in non-English speakers. Yet, solid proof for this is scarce. In order to test the potential learning effects derived from watching subtitled media, a group of intermediate Spanish students of English as a foreign language watched a 1h-long episode of a TV drama in its original English version, with English, Spanish or no subtitles overlaid. Before and after the viewing, participants took a listening and vocabulary test to evaluate their speech perception and vocabulary acquisition in English, plus a final plot comprehension test. The results of the listening skills tests revealed that after watching the English subtitled version, participants improved these skills significantly more than after watching the Spanish subtitled or no-subtitles versions. The vocabulary test showed no reliable differences between subtitled conditions. Finally, as one could expect, plot comprehension was best under native, Spanish subtitles. These learning effects with just 1 hour exposure might have major implications with longer exposure times.

## Introduction

It is often claimed that watching subtitled films and series implicitly helps learning a second language. English is the world’s most widely learnt foreign language [1]; hence the potential of learning through watching subtitled media can have a great economic and social impact (see [2,3] for commercial applications of this principle). Nevertheless, this widespread claim in society lacks conclusive scientific evidence. In fact, the education experts and the public media have mixed opinions about the feasibility of learning languages through watching films with subtitles [4,5]. In many European countries TV shows and other media content are broadcasted in their original version with subtitles (Sweden, Belgium, Denmark or the Netherlands), and even in countries such as Germany, France or Spain where films and foreign media content are regularly dubbed, subtitled audiovisuals in English are becoming increasingly popular because access to audiovisual media in its original language with subtitles is easy. This study aims to investigate the potential benefits of watching subtitled media in major aspects of second language learning such as phonology and vocabulary.

Regarding phonology, the process of learning the sound categories of a second language (L2) as an adult can be a rather challenging task. Often learners have more success learning foreign vocabulary, orthography and syntax than phonology, both in terms of production and understanding [6,7]. This is attributed to the fact that native language perceptual categories are established early in life and are difficult to retune afterwards. One of the problems with acquiring L2 phonology is phonetic variability. The same way people can recognise a familiar face from different angles and lighting conditions, a listener must be able to extract and understand lexical items despite great variability in word form, depending on the acoustic conditions or the speaker’s voice and accent [8]. Indeed, it has been proposed that listeners can shift their native phonetic category boundaries in a flexible way in order to adapt to phonemic variations based on context [8–10]. This re-tuning is especially important to understanding how we learn to distinguish phonemes in foreign languages and adapt to their allophonic variations. Subtitles provide just-in-time written lexical information that can help disambiguate and parse phonemic information [9,11], eventually facilitating the acquisition and/or fine tuning of difficult foreign phonemic categories. Can viewers learn by linking phonological to lexical information while watching subtitled media in a second language? And, if so, what are the most favourable subtitling conditions? Surprisingly, the effects of subtitles in improving perception of second language phonology have received little attention.

Several previous studies have argued that subtitles in audiovisuals can facilitate other aspects of second language learning such as vocabulary acquisition, or overall plot comprehension. Even in these studies, there is controversy regarding the consequences of the different subtitling conditions and the relation to the listener’s proficiency. For instance, Vulchanova et al (2015) claim that both intra and interlingual subtitles (in the language of the soundtrack, or the listener’s native language, respectively) result in improved plot comprehension and vocabulary learning [12]. Some other studies, however, argue that intralingual subtitles are superior than interlingual ones in facilitating content comprehension, vocabulary acquisition [13,14], and second language production [15]. In intralingual subtitles lexical information would allow the listener to link sounds with known word spellings, thus promoting the formation and/or retuning of perceptual categories for a better decoding of speech input. Furthermore, intralingual subtitles help reveal word boundaries (segmentation) and unify accent variations [13]. However, most of the studies mentioned above measure vocabulary (either recall or recognition), general plot comprehension and/or spoken word recognition, but neglect actual speech perception (as in spoken word recognition during natural running speech), where perceptual learning of phonology might be best reflected. Pertinently, a study by Mitterer and McQueen (2009) [16] reported an experiment with Dutch participants proficient in (British) English who listened to audiovisuals in unfamiliar (Scottish or Australian) English accents and afterwards had to repeat back fragments from the dialogue[16]. In their study, intralingual subtitles improved participant’s performance whilst native language (Dutch) subtitles (interlingual) harmed it, compared to a no-subtitle baseline. Based on this finding, Mitterer and McQueen concluded that lexically-guided perceptual learning occurred only when reading original language (English) subtitles.

Complementary to Mitterer and McQueen, who measured whether subtitles helped in accented speech perception with advanced learners, in the present study we address whether watching subtitled English-language films improves English speech perception in intermediate ESL learners. In order to eventually extrapolate these findings to practical applications for language teaching in non-proficient students, we selected an actual TV show where the more conventional (British) English accent is used, and targeted a population of non-proficient second language learners (intermediate to advanced ESL speakers) who find it challenging to understand the target language. This study capitalises on previous findings that subtitles support the disambiguation of perceptual input, found in studies using accented English [16,17], noise vocoded speech [18], ambiguous sounds in a native language [9], or for L2 beginners [19,20]. We address whether this same process might have a further impact on an L2 learning episode, resulting in longer lasting acquisition of phonological knowledge. Also, we hypothesize that intralingual subtitles should enhance top-down, lexically-driven formation of both the speech-sound categories and their adaptation to phonological variations. We will use a listening test to reflect this potential effect.

Above and beyond phonological re-tuning, another important aspect of L2 learning is vocabulary. Some previous studies have found that audiovisuals with subtitles can help acquire new vocabulary [19,21,22]. Written presentation seems to help single out unknown words from the spoken stream [23] and, according to some studies, the integration of sound, video and spelling leads stronger memory trace than visual and audio stimuli alone [24]. Yet, this has not been unequivocally established [25]. We therefore decided to test vocabulary acquisition of the written words in the subtitles.

Finally, an important and practical aspect of subtitles is to facilitate the viewer’s comprehension of the film’s plot. This is in fact, the main purpose of subtitles. Therefore, we addressed overall plot comprehension. The effects of subtitles on overall plot comprehension are not trivial. From the attention literature regarding dual task, one would expect attention competition between reading the subtitles and following the film’s action to be costly in any subtitling condition [26,27]. Focusing attention on quickly presented written text whilst processing the audio and visual content of the scene could compete with the integration of meaning and hence interfere in the overall comprehension of the plot [27] (though see [28]). It is conceivable that this effect could vary between intralingual and interlingual conditions, as reading native vs. foreign languages may lead to different levels of cognitive load, albeit a recent study shows that the fixation time spent in the subtitle area of each condition did not differ” [25]). The plot comprehension test was also used as a way to induce an attentive state and to measure understanding of the participants.

This study was organized in three phases: pre-test, exposure (film watching) and post-test. Participants engaged in all three phases sequentially, within the same session. In both pre- and post-tests, participants were evaluated on their English listening skills and on a vocabulary test. The post-test also included a plot comprehension questionnaire, in addition to the mentioned listening and vocabulary tests.

## Materials and Methods

This project is embedded in the "Lab2Life (From Lab to Life: The impact of multisensory integration phenomena in everyday life)”, which received ethical consent by the ethical committee of the Universitat Pompeu Fabra (CEIC, Parc de Salut Mar, Barcelona, Spain), abiding to the ethical standards of the Declaration of Helsinki.

Sixty university students volunteered for the study. Participants gave written, informed consent after reading about the nature of the study and the future use of the data. They were Romance language speakers (Catalan, Spanish, or Italian) most of them Spanish-Catalan bilingual, between 21–28 years of age, who had studied English through the Catalan school system (thus, after high school, their level is equivalent to B2, following the Common European Framework of Reference for Languages, from the Council of Europe).

Prior to the experimental session, participants took the “face2face written placement test” from Cambridge University [29] (20m duration) in order to assess their English level objectively. Then they were assigned to each condition of the experiment pseudo-randomly, balancing average proficiency of English across groups. During the experimental session, all participants watched the first episode of the 2010 British series ‘Downton Abbey’ (1:08h long) with English subtitles (N = 20), Spanish subtitles (N = 20) or no subtitles (N = 20), depending on the assigned group condition. For the English subtitles, we decided to use non-verbatim subtitles due to the conclusion of the A. Zarei and Rashvand (2011) study [30], which proved that they result in better comprehension. All participants took a listening and vocabulary test before and after watching the episode, and a plot comprehension test only after the episode. The test items in the listening and vocabulary tasks were counterbalanced, so that the particular items of the pre- and post-test were different for a given participant, but on average each item appeared equally across conditions and across pre- and post-test phases. Each test is described in detail, below.

In the listening test participants were asked to fill in 24 word gaps interspersed within a 180 word text, while they were hearing the same text fragment spoken, including the words corresponding to the gaps. The spoken fragment lasted one minute and it was played twice, with one-minute rest in-between repetitions. The 180-word excerpts were taken from a dialogue from episode 2 of the same series, which was held between two new characters that did not appear in the episode viewed by our participants (episode 1) during the exposure phase. This was done in order to maintain a similar language register but avoid the habituation to the particular voice and prosody of the characters featuring in episode one. Orthographic errors in the responses to the listening task were not taken into account (as long as the word was understandable), for only auditory perception was of our interest.

The vocabulary test consisted of 15 definition-matching items in order to capture possible passive acquisition of new words. The target words of the vocabulary test were low frequency English words (approximately 50 times per million words) that appeared in relatively high frequency during the episode (3 to 11 instances). Hence we thought it likely that most subjects did not know these words before and could acquire them during the episode. The word frequency, type, cognation and register or “area of use” was balanced between the two sets of test words (for a more detailed description of the tasks see S2 File).

Finally, in the post-test phase, in addition to the listening and the vocabulary tests, participants responded to 8 comprehension questions about the story they had just watched in order to evaluate their level of attention and comprehension of the episode.

The particular purpose of the study was not revealed to the participants; they were only informed that the study investigated second language acquisition. The counterbalanced design of the tests worked as a control for possible differences between test materials and groups. Half of the subjects were tested with one half of the materials as pre and the other half of the materials as post-test, whilst the rest of participants had the pre- post-test materials reversed. Pilot tests (results not included here) were performed before running the actual experiment (one on a native English speaker and two to Spanish students) in order to adjust parameters and overall duration, and also assess the reliability and sensitivity of the tasks.

The British TV series *Downton Abbey* was first aired in 2010 and the events of the series are set in 1912. The plot focuses primarily on the inheritance of the Estate of Downton, belonging to the aristocratic Grantham family. This series was chosen because of its linguistic characteristics: Standard British (aristocratic twentieth century English), lack of slang and relatively high use of low frequency words. Participants were only included if they declared not having watched the TV series *Downton Abbey* before.

## Results

ListeningVocabComprehListeningVocabComprehTable 1 presents a summary of the results of the study; average scores for pre- and post-tests are shown, with their standard deviation (SD) and their respective percentage (%) over the maximum possible score in the test.

**Table 1:** Descriptive statistics.

		Pre-test			Post-test			Difference		
Test	Group	Average	SD	%	Average	SD	%	Average	SD	%
**Listening**	**None**	9.95	(3.65)	41.46	11.65	(3.50)	48.54	1.70	(1.87)	7.08
	**Eng.**	10.40	(4.33)	43.33	14.45	(4.29)	60.21	4.05	(3.30)	16.88
	**Spa.**	12.20	(4.80)	50.83	12.20	(5.35)	50.83	0.00	(1.69)	0.00
**Vocabulary**	**None**	9.95	(1.99)	66.33	11.25	(1.25)	75.00	1.30	(2.25)	8.67
	**Eng.**	9.30	(2.45)	62.00	10.20	(2.98)	68.00	0.90	(2.83)	6.00
	**Spa.**	10.30	(2.58)	68.67	9.55	(2.72)	63.67	-0.75	(2.17)	-5.00
**Comprehension**	**None**	-	-	-	4.75	(1.55)	59.38	-	-	-
	**Eng.**	-	-	-	6.35	(1.14)	79.38	-	-	-
	**Spa.**	-	-	-	7.45	(0.76)	93.13	-	-	-

**Fig 1:**
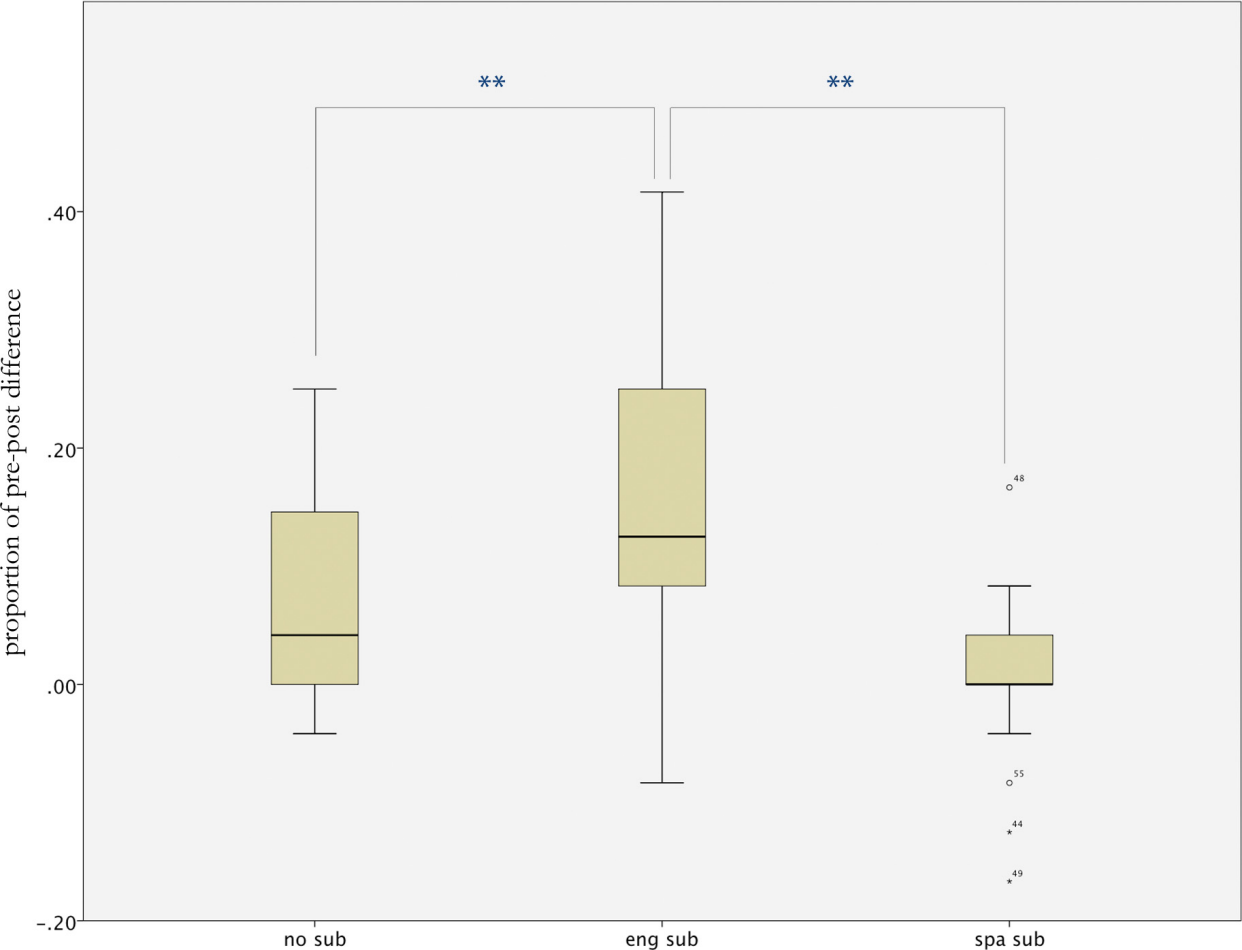
Listening test results. Bonferroni-corrected T-tests for pre-post differences in performance, in the listening test. *<0.05; **<0.01. The English subtitles condition was found to lead to steeper improvements in performance (+4.05 items; 16.9% increase), compared to the no subtitles condition (+1.7 items; 7.1% increase) and to the Spanish subtitles (0.0% increase).

We ran two separate mixed-design ANOVAs using subjects as the random factor (reported as F1), one for the listening test and one for the vocabulary test, with subtitle settings as a between-participant factor (Spanish, English or No subtitles); and pre- and post-test scores as a within-participant repeated measures variable. Additional ANOVAS using items as the random factor were also run (reported as F2). Listening improved significantly from pre- to post-test, that is, after watching the episode (F1_(1.57)_ = 38.786, *p*<0.01, ηp^2^ = 0.405; F2_(1.47)_ = 10.882, *p* = 0.02, ηp^2^ = 0.188). Interestingly, there was an interaction between this exposure effect (difference between pre- and post-test) and the subtitling (F1_(2,57)_ = 14.385, *p*<0.01, ηp^2^ = 0.335; F2_(1.47)_ = 3.710, *p* = 0.06, ηp^2^ = 0.073). Further paired t-tests were then run to evaluate the pre-post differences within each subtitle setting. We found that listening performance improved significantly after watching the film with English subtitles (t1_(19)_ = 5.488, *p*<0.01, *d* = 1.21; t2_(47)_ = 5.555, *p*<0.01, *d =* 0.80) and with No-subtitles condition (t1_(19)_ = 4.073, *p*<0.01, *d* = 0.91; t2_(47)_ = 2.453, *p* = 0.018, *d =* 0.35), but not with Spanish subs (t1_(19)_ = 0.032, *p* = 0.975; *d* = 0.00 t2_(47)_ = 0.513, *p* = 0.610, *d* = 0.07). In order to differentiate between the resultant improvements with English and no-subtitles settings, and to subtract any possible baseline effects arising from group differences, we ran a comparison for the net effect of exposure (pre-post subtraction) between subtitling conditions (Fig 1). Post-hoc (Bonferroni-corrected student t-tests, α = 0.017) revealed that the exposure effect in the English subtitles condition was significantly higher than in the Spanish (t1_(38)_ = 5.345, *p*<0.01, *d* = 1.73; t2_(47)_ = 5.258, *p*<0.01, *d* = 0.69) and the no-subtitles conditions (t1_(38)_ = 3.101, *p*<0.01, *d* = 1.01; t2_(47)_ = 3.484, *p*<0.01, *d* = 0.48). The difference was not significant between Spanish subtitles and no subtitles, though with a marginal trend in favour of the no-subtitles condition (t1_(38)_ = 2.244, *p* = 0.086, *d* = 0.73; t2_(47)_ = 1.926, *p* = 0.06, *d* = 0.24). Thus, importantly, the English (intralingual) subtitles led to a clear improvement in the listening test, which was not seen with Spanish subtitles. Interestingly, a weaker but significant improvement was also seen without subtitles.

Regarding the vocabulary task, the mixed-ANOVA showed no overall pre-post improvement (F1_(1,57)_ = 2.344, *p* = 0.131, *ηp*^*2*^ = 0.039; F2_(1,29)_ = 1.375, *p* = 0.251 *ηp*^*2*^ = 0.045), but the interaction was significant (F1_(2,57)_ = 4.011, *p = 0*.023, *ηp*^*2*^ *= 0*.123; F2_(1,29)_ = 6.313, *p =* 0.018, *ηp*^*2*^ *=* 0.179). In the follow-up t-tests on the net pre-post difference scores, we found that both the English subtitles and no-subtitles condition revealed a modest pre-post improvement (6% and 8.7% respectively), though only significant in the no-subtitles condition (English: t1_(19)_ = 1.407, *p* = 0.175; *d* = 0.32; t2_(19)_ = 1.373, *p* = 0.18; *d* = 0.25; no subtitles t1_(19)_ = 2.584, *p* = 0.018; *d* = 0.59; t2_(29)_ = 2.300, *p* = 0.029; *d* = 0.44). The Spanish subtitles condition showed no pre-post difference (t1_(19)_ = -1.564, *p* = 0.134; *d* = 0.35; t2_(29)_ = -1.286, *p* = 0.205; *d* = 0.24). The interaction was then likely a result of weak effects in opposed directions (8.6% and 6% improvements in the no-subtitle and English subtitle condition, combined with a non-significant 5% pre-post decrement in the Spanish subtitle condition). In fact, when relative pre-post differences were compared using paired t-tests between conditions, none of the paired comparisons survived the significance criterion (Bonferroni corrected α = 0.017).

Finally, a one-way ANOVA was run on the data from the plot comprehension test, showing significant differences between subtitle conditions (F1_(2.57)_ = 25.863, *p*<0.01, ηp^2^ = 0.476; F2_(2,23)_ = 6.824, *p*<0.01, ηp^2^ = 0.544). Bonferroni post-hoc test (α = 0.017) showed this effect displayed a graded pattern where Spanish (interlingual) subtitles led to better comprehension scores than the English subtitles condition (t_(38)_ = 2.913, *p* = 0.015, *d* = 0.94), and the English (intralingual) subtitles led to better comprehension than the no-subtitles condition (t_(38)_ = 4.237, *p<*0.01, *d* = 1.37). Trivially, Spanish subtitles where superior to no subtitles (t_(38)_ = 7.151, *p<*0.01, *d* = 2.32). Please note that the item analysis is unreliable here, since there were only 8 items in the test and therefore, it will not be reported.

Please note percentage scores might be subject to distortion in parametric tests due to low variability in the extremes of the distributions. Despite our data being generally far from these extremes, we repeated all of the above analyses using a log odds transformation of the proportion scores and confirmed the main pattern of results, described above (see S1 File).

## Discussion

The most relevant finding to emerge from this study was a significant improvement in listening scores of ESL speakers, after watching a TV episode in English, with English subtitles (17% increase vs. 7% for no-subtitles and 0% for Spanish subtitles condition). This finding supports the hypothesis that subtitles in the original language can be used to retune the link of speech-sounds with perceptive categories, so that intermediate to advanced English learners can adapt to English sounds in a more efficient fashion.

In addition, we observed that simply watching the episode with the original English soundtrack without any subtitles already produced a modest, but statistically significant improvement in the listening test. This more subtle effect may have been a consequence of the ability of our intermediate ESL participants to capitalise on sufficient information in the no-subtitle condition, together with the fact that the listening task was relatively challenging, leaving good room for improvement (results for the listening task varied from 30% to 50%). Ultimately, the Spanish subtitles resulted in the lowest post-test listening scores, and no pre-post differences. This pattern of results is in partial agreement with the results reported by Mitterer and McQueen’s (2009), because we replicated the advantage conferred by intralingual subtitles, yet instead of a performance detriment in the interlingual subtitles condition we found that Spanish subtitles simply blocked improvement (i.e., the potential learning undergoing by simply watching the show without subtitles disappeared). Possibly, the difference in language proficiency of the participants (more proficient in Mitterer and McQueen’s) could explain the different results. However, Mitterer and McQueen’s effect was small (N = 40, *p* = 0.05, *d* = 0.35), hence it is possible that it would show under the present conditions if we had a higher number of participants per group.

Regarding vocabulary acquisition, our results showed modest pre-post improvements following the English (6%) and no subtitle viewing conditions (8.7%), though only significant in the no-subtitles condition. Yet, none of these pre-post differences were different from each other in the paired comparison post-hoc test. Altogether, these results do not show conclusive evidence for a clear acquisition of new vocabulary after watching the episode, and no modulation due to the subtitles’ condition. This goes against our initial hypothesis and some previous findings showing vocabulary acquisition with intralingual subtitles ([21,22], although see [25] for a similar failure to acquire new vocabulary watching foreign language films). Perhaps this lack of effect was due to the difficulty of the material, mainly composed of low frequency words, many from the twentieth century aristocracy, with a mean of 5 repetitions in the episode. We will treat this particular result carefully in our final conclusions.

On the subject of plot comprehension, viewers of the Spanish subtitles condition performed significantly higher than those in the English condition who, in turn, outperformed participants who watched the TV show without subtitles, as expected. It is clear that the opportunity to read Spanish subtitles obviously benefited overall comprehension due to the language proficiency advantage. Furthermore, in accordance with previous literature [24,31] our results show that reading subtitles whilst processing visual and auditory input does not seem to compete with the actual integration of meaning, hence it did not affect plot comprehension.

Nowadays, we often have the chance to watch audiovisual media in the original language, with the option of overlaying subtitles. The current study confirms that lexically-driven perceptual learning occurs while viewing subtitled English audiovisuals, in a naturalistic setting of subtitled TV series. Hence, under these conditions, quick adaptation to a second language (here, Standard British English) is possible for intermediate to advanced English students with English subtitles, leading to an improvement in their listening skills. A practical implication to real life can be tentatively extrapolated from these findings: Once a learner has reached the intermediate to advanced level of a language, the best condition for improving speech perception whilst watching series or films, is listening to the original soundtrack with subtitles in the original language. Despite this is a rather popularised claim, empirical evidence was lacking. Unlike with listening skills, the present data did not conclude that there was beneficial acquisition of new vocabulary in any of the conditions. If anything, there were weak trends toward vocabulary improvement after watching the TV series without subtitles or English subtitles, but not with Spanish subtitles.

Another important conclusion of our study for practical applications is that the putatively beneficial effects of intralingual subtitles come at a cost in plot comprehension, compared to subtitles in the viewer’s own language (interlingual). However, it is remarkably positive that English subtitles did not add sufficient cognitive load as to disrupt overall plot comprehension due to dual task costs, as plot comprehension with English subtitles still outperformed plot comprehension without subtitles. Although this is a small effect that will need further investigation, please note that it was obtained in naturalistic film watching conditions, equivalent to how one would watch a TV show at home. From these results, viewers need to evaluate the trade-off between the level of plot understanding and the beneficial effects for their L2 learning, as our results show comprehension costs for the no subtitles and English subtitled conditions in comparison with Spanish subtitled or, as an extension, dubbed material.

## Supporting Information

S1 File LogOdds transformation of the proportions; results and tables. (DOCX)

S2 File Tasks’ description. (DOCX)
