# Supplementary material for: Watching Subtitled Films Can Help Learning Foreign Languages
Source: PLoS One. 2016 Jun 29;11(6):e0158409. doi: 10.1371/journal.pone.0158409 (PMC4927148; doi:10.1371/journal.pone.0158409)
Supplement: S1 File — (DOCX) [file pone.0158409.s001.docx]

**Supporting Information – S1 File**

J. Birulés-Muntané, S. Soto-Faraco

**Description of supporting information files**

- S1 File. LogOdds transformation of the proportions; results and tables
  - **Table A**: T-tests for pre-post differences with Log Odds data (both for subjects and participants)
  - **Table B**: Bonferroni-corrected T-tests for pre-post differences with proportionns and LogOdds transformed data

**LogOdds transformation of the proportions; results and tables**

The data was transformed using LogOdds transformation of the proportion (log p/1-p). The analysis with the transformed data replicated the results for the mixed-design ANOVA of the Listening task, showing a pre-post improvement (F1_(1,57)_=16.576, *p*<0.01, *ηp^2^*=0.225; F2_(1,47)_=10.995, *p*<0.01, *ηp^2^*=0.190) and a condition-pre-post interaction (F1_(1,57)_ =8.280, *p*<0.01, *ηp^2^*=0.239; F2_(1,47)_=2.115, *p*=0.153, *ηp^2^*=0.043).

In the vocabulary task, there were no pre-post differences, (F1_(1,57)_= 2.578, *p*=0.114, *ηp^2^*=0.043; F2_(1,29)_=0.999 *p*=0.326, *ηp^2^*=0.033) and only a tendency for the interaction (F1_(1,57)_= 3.023, *p*=0.057, *ηp^2^*=0.096; F2_(1,29)_=8.900, *p*<0.01, *ηp^2^*=0.235)

Following, Table A shows the T-test results for Listening and Vocabulary, and Table B reports the Bonferroni post-hoc test for the three tasks.

**Table A** T-tests for pre-post differences with Log Odds data (t1 and t2)

|  |  | **T1 Log Odds** | | **T2 Log Odds** | |
| --- | --- | --- | --- | --- | --- |
| **Test** | **Group** | **t-value** | **p-value** | **t-value** | **p-value** |
| **Listening Task** | **English** | -3,898 | <0.01 | -5.525 | <0.01 |
|  | **Spanish** | 0.484 | 0.634 | -0.594 | 0.556 |
|  | **No** | -3.892 | <0.01 | -2.242 | 0.03 |
| **Vocabulary Task** | **English** | -1.896 | 0.073 | -1.224 | 0.231 |
|  | **Spanish** | 0.945 | 0.357 | 1,882 | 0.07 |
|  | **No** | -2.558 | 0.019 | -2.406 | 0.023 |

**Table B.** Bonferroni-corrected T-tests for pre-post differences with proportions and LogOdds transformed data.

|  |  |  | **Proportions** | **Log Odds** | **Items- Log Odds** |
| --- | --- | --- | --- | --- | --- |
| **Test** | **Group (i)** | **Group (j)** | **p-value** | **p-value** | **p-value** |
| **Listening** | **No** | **English** | .009 | .042 | .001 |
|  |  | **Spanish** | .086 | .303 | .153 |
|  | **English** | **No** | .009 | .042 | .001 |
|  |  | **Spanish** | .000 | .000 | .000 |
|  | **Spanish** | **No** | .086 | .303 | .153 |
|  |  | **English** | .000 | .000 | .000 |
| **Vocabulary** | **No** | **English** | 1.000 | 1.000 | .421 |
|  |  | **Spanish** | .030 | .190 | .006 |
|  | **English** | **No** | 1.000 | 1.000 | .421 |
|  |  | **Spanish** | .109 | .074 | .020 |
|  | **Spanish** | **No** | .030 | .190 | .006 |
|  |  | **English** | .109 | .074 | .020 |
| **Comprehension** | **No** | **English** | .000 | .009 |  |
|  |  | **Spanish** | .000 | .000 |  |
|  | **English** | **No** | .000 | .009 | . |
|  |  | **Spanish** | .015 | .097 |  |
|  | **Spanish** | **No** | .000 | .000 |  |
|  |  | **English** | .015 | .097 | . |

Please note the comprehension test has only 8 items and hence the item analysis is not reported. Also note the p- values for the item analysis here come from paired t-test comparisons, since there is no “between factor” in the items data.
