# Supplementary material for: Watching Subtitled Films Can Help Learning Foreign Languages
Source: PLoS One. 2016 Jun 29;11(6):e0158409. doi: 10.1371/journal.pone.0158409 (PMC4927148; doi:10.1371/journal.pone.0158409)
Supplement: S2 File — (DOCX) [file pone.0158409.s002.docx]

**Supporting Information – S2 File**

J. Birulés-Muntané, S. Soto-Faraco

**Description of supporting information files**

- **S2 File.** Tasks’ description
  - **Text A:** Listening task
  - **Text B:** Vocabulary task
  - **Text C:** Comprehension task

**Text A. Listening task**

Chosen words were broadly counterbalanced between tests 1 and 2 by type, number of repetitions (in the episode) and frequency per million. All the definitions were taken from the Longman dictionary of contemporary English, and the wrong answers came from other words. Of the two wrong definitions, one was chosen from inside the semantic field and the other from outside, whenever possible. Word frequencies were taken from the book: “Word Frequencies in Written and Spoken English: based on the British National Corpus” of Geoffrey Leech et al, 2001.

Listening

1. We
2. Are
3. Good
4. Ill
5. Refuse
6. Inherit
7. Throw
8. Up
9. To
10. Butler
11. Valet
12. Mr.
13. Mathew
14. Hand
15. Change
16. Me
17. Unwelcome
18. Heir
19. Lawyer
20. Upper
21. Turning
22. Me
23. Own
24. Met

Listening 2 words:

1. Better
2. Done
3. Ma’am
4. Taken
5. Up
6. Whole
7. Household
8. Kitchen-maid
9. Ridiculous
10. He
11. Valet
12. Maid
13. Behave
14. Confirm
15. Expectation
16. Use
17. Myself
18. Earth
19. Mean
20. Push
21. At
22. Me
23. Fixed
24. Bachelor

Example:

-Mother, Lord Grantham has made the *___(unwelcome)_____* discovery that his *__(heir)_______* is a middle-class ___(*lawyer*)_ and the son of a middle class doctor.

**Listening 1**

- Here __1__ ___2__, ma'am, Crawley House.

- For *__3______*or*___4______*.

- I still don't see why I couldn't just ______5_____ it.

- There's no mechanism for you to do so. You will be an earl. You will *_____6_____*the estate*.* Of course, you can _____7_____it away when you have it. That's __8_ __9__ you.

-Can I help?

-I'm Molesley, sir, your ____10____ and*____11______.*

- Mr Molesley, I'm afraid//

- May I introduce myself? I am ____12_____ Crawley and this is my son, Mr ______13_______ Crawley.

- I'll just give *–*Mr. Taylor a_____14_____with the cases.

- I can//

- Thank you, Molesley.

- I won't let them *___15______ __16___.*

-Why would they want to?

-Mother, Lord Grantham has made the *______17______* discovery that his *___18___* is a middle-class ______19_____ and the son of a middle class doctor.

-____20_____ middle class.

-He wants to limit the damage by ___21____ __22___ into one of his __23___ kind*.*

-When you ___24____ him in London, you liked him.

**Listening 2**

- Oh, Ellen. This is much *___1____*than I thought it would be. You have *___2____*well.

- Thank you, _____3______.

- Would you like this in here, ma'am, or _*_____4___ __5___* to your room?

- In here, thank you.

- So, are you the *____6______*of our new*________7______*?

- There's a local girl, ma'am, Beth. She's to double under-housemaid and ________ ___8_____.

- This is ________9_______.

- Thank you very much, Molesley.

- Might we have some tea?

- Very good, ma'am.

- Well, ___10___ can go right now.

- Why?

-Because we don't need a butler, or a______11______, if it comes to that.

- We've always managed perfectly well with a cook and a *____12_____*, and they cannot expect us to alter our...

-What they expect, Matthew, is that we don't know how to _____13_______. So if you don't mind, I would rather not*_____14_______*their*______15_______.*

- I have to be myself, Mother. I'll be no *__16___*to anyone if I can't be *______17______*. And before they or you get any ideas, I will choose my own wife.

-What on _*__18______*do you*_____19_________*?

- Well, they're clearly going to *___20_____*one of the daugthers *__21____ _22___.*

They'll have ____23____ on that when they heard I was a ____24_______.

**Text B. Vocabulary task**

None of the words is cognate with Catalan or Spanish, and they were balanced by number of repetitions and frequency per million. All the definitions were taken from the Longman dictionary of contemporary English, and the wrong answers came from other words. Of the two wrong definitions, one was chosen from inside the semantic field and the other from outside, whenever possible.

Word frequencies were taken from the book: “Word Frequencies in Written and Spoken English: based on the British National Corpus” of Geoffrey Leech et al, 2001”.

Example:

**EAGER**

a/ to be reasonable, practical, and showing good judgment

b/ fairly or to some degree

c/ very keen and excited about something that is going to happen or about something you want to do

**Test 1**

**1- LORDSHIP**

a/ a man who has a rank in the aristocracy.

b/ a long narrow boat that is used for travelling on a canal

c/ used when talking to or talking about a lord, or when talking to a judge or bishop

**2- FOOTMAN**

a/ someone whose job is to clean other people's houses.

b/ a male servant in the past who opened the front door, announced the names of visitors etc

c/ unable to walk properly because your leg or foot is injured or weak.

**3- SHAME**

a/ to produce bright light

b/ caring and feeling sorry about someone's problems

c/ the feeling you have when you feel guilty and embarrassed.

**4- OUGHT TO**

a/ to write a letter to someone

b/ to make a particular situation exist or cause a particular feeling

c/ to tell someone something because it is the best or most sensible thing to do

**5- HEIR**

a/ the mass of things like fine threads that grows on your head.

b/ the chief male servant of a household, usually in charge of serving food, the care of silverware, etc.

c/ the person who has the legal right to receive the property or title of another person when they die.

**6- INDEED**

a/ fairly or to some degree

b/ used to emphasize a statement or answer:

c/ wanting something that someone else has

**7- SHALL**

a/ to give especial attention or importance to something

b/ used to make a suggestion, or to say what you will do in the future,

c/ to say that you admire and approve of someone or something, especially publicly

**8- ODD**

a/ unable to walk properly because your leg or foot is injured or weak.

b/ different from what is normal or expected, especially in a way that you disapprove of or cannot understand

c/ something exciting that provides information that you did not know about

**9- LUNCHEON**

a/ a room where you eat meals in a house or hotel

b/ formal for lunch

c/ food cooked or prepared in a particular way.

**10- the WAGE**

a/ money you earn that is paid according to the number of hours, days, or weeks that you work

b/ an area where houses or buildings of a similar type have all been built together in a planned way

c/ A treaty or deal of inheritance.

**11- EAGER**

a/ to be reasonable, practical, and showing good judgment

b/ fairly or to some degree

c/ very keen and excited about something that is going to happen or about something you want to do

**12- To GATHER**

a/ to believe that something is true because of what you have seen or heard

b/ to tell someone that you are sorry that you have done something wrong

c/ to regard something as unlikely to be true or important

**13- UNSINKABLE**

a/ impossible to go down below the surface of water or mud

b/ impossible to accept or imagine

c/ something that cannot be eaten

**14- BEG**

a/ move towards a place or position that is in front of you

b/ to put in order; arrange in a desired state or condition.

c/ to ask for something in an anxious or urgent way, because you want it very much

**15- PRY**

a/ to remain hidden because someone is trying to find you or catch you

b/ to try to find out details about someone else's private life in an impolite way:

c/ to talk too proudly about your abilities, achievements, or possessions

**Test 2**

**1- ESTATE**

a/ an area where houses or buildings of a similar type have all been built together in a planned way

b/ the physical or mental condition that someone or something is in

c/ the government or political organization of a country

**2-VALET**

a/ a male servant who looks after a man's clothes, serves his meals etc.

b/ someone whose job is to clean other people's houses.

c/ to be reasonable, practical, and showing good judgment

**3- RATHER**

a/ fairly or to some degree

b/ used to make a suggestion, or to say what you will do in the future:

c/ caring and feeling sorry about someone's problems

**4- MOURNING**

a/ a flat hard area near a house, where people sit outside.

b/ to show everyone how rich you are.

c/ expression of great sadness because someone has died.

**5- DUTY**

a/ something that you have to do because it is morally or legally right

b/ a physical kind of work

c/ someone who prepares and cooks food as their job

**6- ENTAIL**

a/ the area of land next to a house, where there are flowers, and often a place for people to sit

b/ the person who has the legal right to receive the property or title of another person when they die.

c/ a treaty or deal of inheritance.

**7- SETTLED**

a/ to put in order; arrange in a desired state or condition

b/ in a lower place or position

c/ not tidy, planned, or arranged in order

**8- APOLOGISE**

a/ to make it possible or easier for someone to do something

b/ the feeling you have when you feel guilty and embarrassed.

c/ to tell someone that you are sorry that you have done something wrong

**9- GIVE A CHANCE**

a/ to ask for mercy

b/ to give the legal right

c/ to ask for an opportunity

**10- To WONDER**

a/ to walk or travel, usually for a long time, with no clear purpose or direction

b/ to believe that something is true because of what you have seen or heard

c/ to think about something that you are not sure about and try to guess what is true, what will happen etc

**11- DRAWING ROOM**

a/ a room, especially in a large house where you eat meals

b/ a room, especially in a large house where you normally sleep

c/ a room, especially in a large house, where you can entertain guests or relax

**12- FIANCE**

a/ a man who has a rank in the aristocracy, especially in Britain

b/ the man whom a woman is going to marry

c/ a large plate from which food is served

**13- for someone's SAKE**

a/ to go instead of someone, or as their representative.

b/ in order to help, improve, or please someone.

c/ with desire to cause damage on someone.

**14- LAME**

a/ unable to walk properly because your leg or foot is injured or weak:

b/ the daughter of your brother or sister

c/ morally wrong or evil

**15- BUTLER**

a/ the chief male servant of a household, usually in charge of serving food, silverware, etc.

b/ a piece of cloth used for a particular purpose

c/ a man who usually wears a uniform and works in a big house or hotel, letting people into the building

**Text C. Comprehension task**

1. **Perquè no li volen llegir a la Mary la notícia del diari?**

*Porque no quieren leerle a Mary la notícia del diario? Why do they keep the paper news from Mary?*

1. **De qui prové la fortuna originàriament?**

*De quien proviene la fortuna originariamente? Whose is the fortune from, originally?*

1. **Perquè el Duque persuadeix a la Mary a pujar als atics de la casa?**

*Porque el Duque persuade a Mary a subir a los áticos de la casa? Why does the Duke persuade Mary to go to the attics?*

1. **Perquè el Duque marxa el matí següent?**

*Porque se va el Duque la mañana siguiente? Why does the Duke part the following morning?*

1. **Perquè es sorprenen els servents quan el pare saluda al "valet" nouvingut?**

*Porque se sorprenden los sirvientes cuando el padre saluda al nuevo "valet"? Why do servants get surprised when the father greets the new valet?*

1. **Perquè en Thomas (servent) li fa la vida impossible al nouvingut?**

*Porque Thomas el sirviente le hace la vida imposible al nuevo valet? Why is Thomas making the valet’s life imposible?*

1. **A què es refereix l'última frase: Ens vol canviar les vides?***A qué se refiere la última frase: nos quiere cambiar las vidas? What does the last sentence refer to: “He wants to change our lives”*
2. **Què és tant preocupant al llegir el diari/la** carta al començament del capítol? *Qué es tan preocupante al leer el diario/carta al principio del capítulo? What’s so worrying about the newspaper, in the beginning of the chapter?*
